# Navigating Diagnostic Challenges: Heterotopic Pregnancy Versus Hyperdecidual Reaction in Ectopic Gestation

**DOI:** 10.7759/cureus.61073

**Published:** 2024-05-25

**Authors:** Ipsita Mohapatra, Subha R Samantaray

**Affiliations:** 1 Obstetrics and Gynecology, All India Institute of Medical Sciences, Bhubaneswar, Bhubaneswar, IND; 2 Obstetrics and Gynecology, All India Institute of Medical Sciences, Kalyani, Kalyani, IND

**Keywords:** heterotopic pregnancy, radiological misdiagnosis, diagnostic challenge, intrauterine pregnancy, hyperdecidual reaction, ectopic pregnancy

## Abstract

In the intricate field of obstetrics and gynecology, few scenarios present as complex a diagnostic challenge as the differentiation between heterotopic pregnancy, hyperdecidual reaction, and ectopic pregnancy. These conditions, while distinct, often blur together in clinical presentation, necessitating a nuanced understanding to achieve accurate diagnosis and timely intervention. A heterotopic pregnancy is a rare and potentially life-threatening condition in which a woman simultaneously carries two pregnancies in different locations. One pregnancy is typically located within the uterus (an intrauterine pregnancy), while the other is located outside the uterus, most commonly in one of the fallopian tubes (an ectopic pregnancy). This condition is sometimes referred to as a combined intrauterine and extrauterine pregnancy. The diagnosis of heterotopic pregnancy can be challenging because the symptoms can mimic those of a normal intrauterine pregnancy or an ectopic pregnancy. A combination of clinical symptoms, physical examination, and imaging studies, such as transvaginal ultrasound, can help in the diagnosis. After surgical or medical treatment, close monitoring and follow-up with a healthcare provider are essential. The remaining intrauterine pregnancy will need careful observation to ensure it continues to develop normally. However, in some cases of ectopic pregnancy, there will be hyperdecidual reaction within the uterus, which may sometimes create confusion with intrauterine pregnancy. Here, a case of ectopic pregnancy that was radiologically misdiagnosed as heterotopic pregnancy is presented to highlight the possibility of ectopic pregnancies being misdiagnosed as heterotopic pregnancy due to the hyperdecidual reaction. The index case underwent laparoscopic salpingectomy for tubal ectopic and dilatation and evacuation for suspected failed intrauterine pregnancy. The histopathological report of the intrauterine products of conception confirmed it to be decidua without any trophoblastic tissue.

## Introduction

Ectopic pregnancy refers to the implantation of a fertilized ovum outside the uterine cavity, commonly occurring in the fallopian tubes [1]. This condition poses significant risks to maternal health, including hemorrhage and loss of fertility if not promptly identified and managed.

Heterotopic pregnancy is the name given to twin pregnancy in which one is intrauterine and the other is extrauterine [2]. It is the pathological form of a bi-ovular dizygotic twin pregnancy in which one embryo gets implanted in the normal uterine cavity and the other one gets implanted outside the uterine cavity [3]. The cause may be a defect in ovulation or a defect in the fallopian tube to catch the fertilized egg [3]. The incidence of heterotopic pregnancies due to artificial reproductive techniques has increased in the recent past and is about 1.5 per 1000 to 1% [4,5]. However, the incidence of spontaneous heterotopic pregnancy is only about one in 30,000 pregnancies [6]. The diagnosis is often late due to the lack of suspicion and hence often diagnosed after rupture of the ectopic pregnancy. The management goal is to remove the ectopic pregnancy without disturbing the intrauterine pregnancy and hence always difficult.

Hyperdecidual reaction refers to the exaggerated decidualization of the endometrium, mimicking early intrauterine gestation. This phenomenon can occur in ectopic pregnancies, complicating their diagnosis by simulating the presence of an intrauterine pregnancy on ultrasound examination.

Here, we present a case of ectopic pregnancy that was misdiagnosed as heterotopic pregnancy, its course of management, and follow-up.

## Case presentation

**Figure 1:**
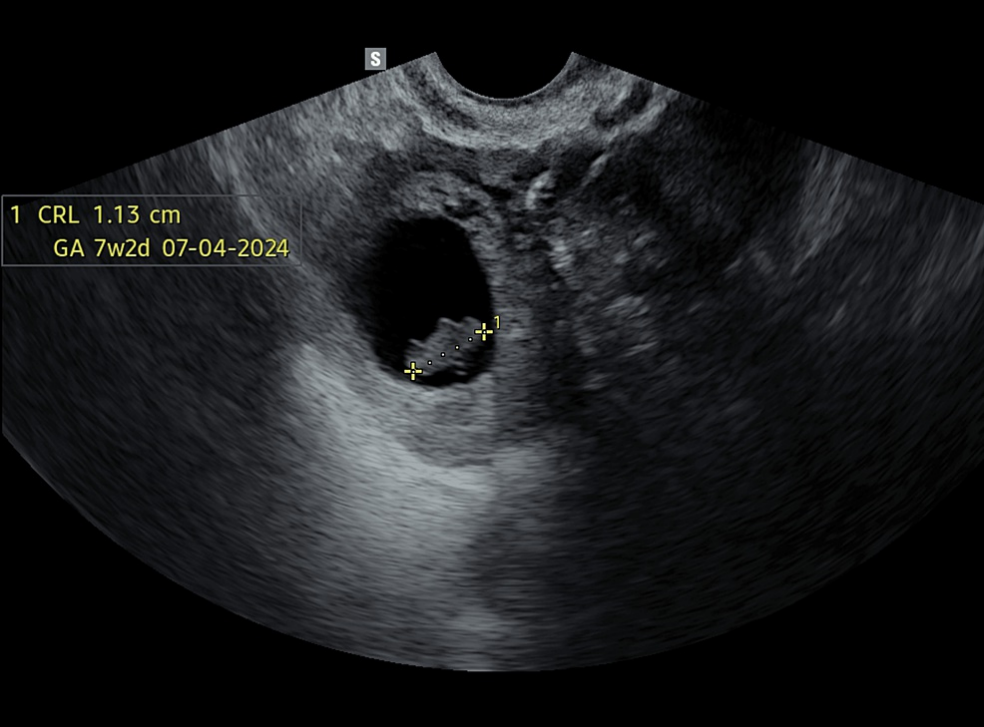
Tubal pregnancy CRL: crown-rump length, GA: gestational age

A 31-year-old lady came to the gynecology outdoor clinic with the chief complaint of primary infertility. She was married for four years and was unable to conceive in spite of regular unprotected intercourse. On assessment of her menstrual history, she was found to have irregular cycles. There was no abnormality detected in her sexual history. Based on the history, investigations were ordered in the line of infertility like serum anti-Mullerian hormone, thyroid profile, prolactin, luteinizing hormone, follicular-stimulating hormone, histo-salpingography, and husband's semen analysis. Her serum prolactin levels were raised (56 mIU/L), and serum AMH was low (0.8ng/ml). On her next visit, she gave a history of amenorrhea for five weeks, and her urine pregnancy test came out to be positive. This was a spontaneous pregnancy. She was ordered a transvaginal ultrasonography for dating of pregnancy. The report showed very early intrauterine pregnancy. The patient was started on micronized progesterone support, and folic acid supplementation was continued. A second transvaginal ultrasonography was ordered at eight weeks of amenorrhea duration. The report suggested it to be a heterotopic pregnancy with one intrauterine empty gestational sac of 3 cm diameter and another gestational sac in the right fallopian tube. The tubal pregnancy was unruptured with a sac diameter of 2.7 cm. The crown-rump length (CRL) was 1.13 cm (Figure 1).

**Figure 2:**
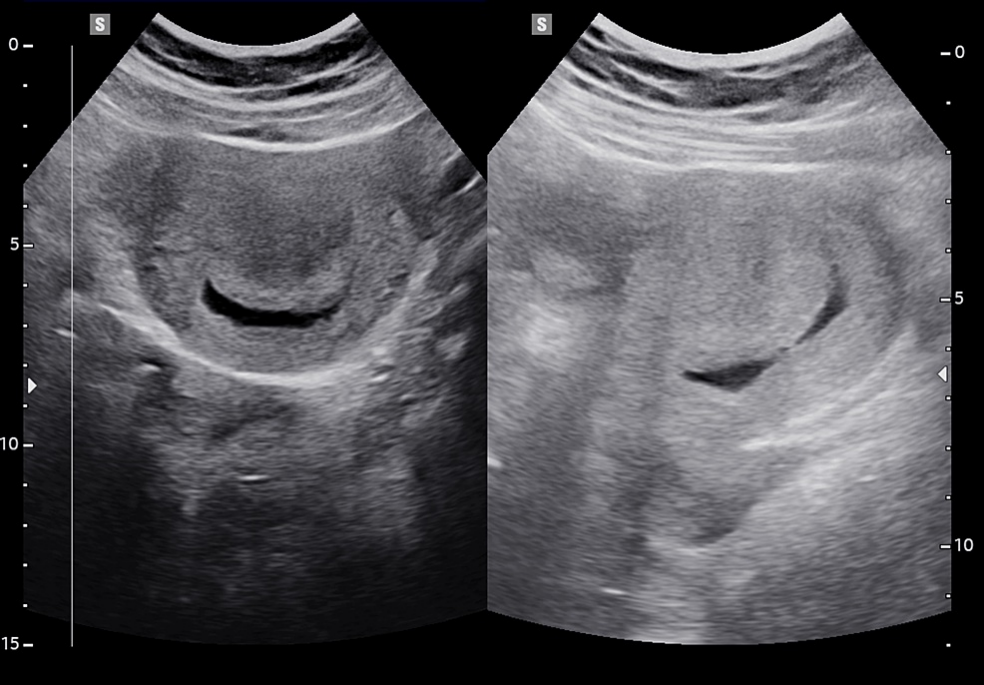
Hyperdecidual reaction misinterpreted as an empty intrauterine gestational sac

The intrauterine pregnancy revealed an empty gestational sac (Figure 2).

A plan was taken for surgical intervention, and the patient was posted for laparoscopic salpingectomy. Laparoscopic salpingectomy was done, and in the same setting, dilatation and evacuation of the intrauterine pregnancy were done in view of the empty gestational sac. The histopathological report revealed the presence of decidua and confirmed the absence of any chorionic tissue. Thus, the final diagnosis was right tubal ectopic pregnancy with intrauterine hyperdecidual changes.

## Discussion

The presentation of these conditions often overlaps, confounding clinicians and necessitating a multifaceted approach to diagnosis. Patients may present with pelvic pain, vaginal bleeding, and signs of intra-abdominal hemorrhage, which are hallmarks of both ectopic and heterotopic pregnancies. Hyperdecidual reaction is an exaggerated decidual response of the endometrial lining, typically in response to ectopic pregnancy, hormone therapy, or early miscarriage. It can mimic the appearance of an early intrauterine pregnancy on ultrasound, leading to potential misdiagnosis. The presence of a hyperdecidual reaction can mimic the signs of early intrauterine gestation in imaging studies, adding another layer of complexity to the diagnostic process. When a hyperdecidual reaction occurs, the endometrial lining thickens and forms a pseudo-sac. This pseudo-sac can resemble a gestational sac seen in early intrauterine pregnancy, leading to misdiagnosis. Ectopic pregnancy is a life-threatening obstetrics emergency in the early trimester, associated with high morbidity and mortality if not intervened timely [7], which may happen in cases of misdiagnosis due to the hyperdecidual reaction.

Heterotopic pregnancy was once considered a rare entity. However, due to the increase in late age at the first conception, increase in the rate of pelvic inflammatory diseases, and increased number of couples conceiving through assisted reproductive techniques, the incidence of ectopic and heterotopic pregnancies has increased. However, the incidence of spontaneous heterotopic pregnancies is only about one in 30,000 pregnancies [6]. The risk factors for heterotopic pregnancies are similar to those of ectopic pregnancies, like history of previous ectopic pregnancy, pelvic inflammatory diseases, sexually transmitted diseases, prior surgeries on the fallopian tube, smoking, infertility treatment, any other abdominal surgery, and endometriosis. Patients with no risk factor account for only a few cases of heterotopic pregnancies [2].

The most common site of ectopic pregnancy is the fallopian tube (95-97%). Of these, most are located in the ampullary region (80%), while the others are in the isthmic (10%), fimbrial (5%), and corneal and interstitial regions (2-4%) [8]. The case presented here was implanted in the interstitial portion of the fallopian tube.

The patients with spontaneous heterotopic pregnancy (SHP) typically present with symptoms of abdominal pain, vaginal bleeding, and spotting, and in severe cases, they may present with acute abdomen, shock, and haemo-peritoneum similar to those with ectopic pregnancy. Some patients like the index case may be asymptomatic also. All these patients have a history of amenorrhea followed by these symptoms. The diagnosis is often late because the presence of intrauterine pregnancy gives a false reassurance and most of these symptoms point toward signs of threatened or pregnancy loss. A systematic review observed that about 33% of heterotopic pregnancies were misdiagnosed due to a normal first-trimester scan [2]. The common differential diagnoses of SHP are miscarriage, ectopic pregnancy, intrauterine pregnancy with hemorrhagic corpus luteum, adnexal torsion, and non-gynecological causes, like appendicitis, cholecystitis, bowel obstruction, or pancreatitis [9].

The exact prevalence of a hyperdecidual reaction misdiagnosis is not known, but it may be more in the early gestational period due to a similar appearance before fetal poles are seen. Elevated hormone levels like progesterone, mainly in the cases of fertility treatment, may be one of the causes of hyperdecidual intrauterine changes

The serum β-HCG levels are also not diagnostic. In the case of ectopic pregnancies, there is a failure of serum β-HCG to rise by 33% in 48 hours [10]. However, in the case of SHP, the concept of doubling time and discriminatory level of 3500 iu/ml is not helpful due to the presence of simultaneous intrauterine pregnancy leading to misdiagnosis of heterotopic pregnancy. Ultrasonography is the first and preferred method of diagnosis. MRI can be done in selected patients in the case of doubtful diagnosis.

Any form of treatment in the case of heterotopic pregnancy is aimed at the ectopic pregnancy selectively so that the intrauterine pregnancy is not disturbed. The most common treatment for a heterotopic pregnancy is surgical intervention. This typically involves removing the ectopic pregnancy while preserving the intrauterine pregnancy, if possible. The specific surgical approach may vary depending on the location and size of the ectopic pregnancy. It could involve laparoscopy (minimally invasive surgery) or laparotomy (open surgery). Salpingectomy or salpingotomy may be done, but salpingotomy is usually not preferred due to the risk of continuous tubal bleeding. Salpingotomy also carries the additional risk of persistent trophoblasts in the fallopian tube, which is difficult to follow up by serum β-HCG due to the continuing intrauterine pregnancy [11]. In patients with an intact contralateral tube, fertility results after salpingectomy are almost equal to those after salpingotomy [12]. Whatever may be the procedure undertaken, minimal intraoperative manipulation of the uterus is advised in order to prevent rupture and damage to the intrauterine pregnancy [13].

In some cases, if the ectopic pregnancy is small and has not yet ruptured, it may be possible to treat it with medication, such as methotrexate. Local injection of feticidal agents like potassium chloride and hyperosmolar glucose under transvaginal guidance can also be considered for termination of the ectopic pregnancy [14]. However, this approach is usually not preferred due to the systemic side effects and bad prognosis of intrauterine pregnancy, high chances of failure of medical management, and risk of rupture of the ectopic pregnancy resulting in emergency surgery [15]. A greater risk of abortion of the intrauterine pregnancy is observed in patients undergoing medical versus surgical management at a rate proportion of 50%:13% [16].

The misdiagnosis of hyperdecidual reaction as intrauterine pregnancy can be minimized by correlating the clinical findings and serum β-HCG levels with the ultrasound findings. Serial ultrasound images will show progressive development of fetal poles in case of intrauterine pregnancy, which was not seen in the index case raising the suspicion towards intrauterine hyperdecidual changes. A detailed ultrasonography should be done to find the location of the sac. A pseudo-sac typically lacks the double decidual sac sign, which is a reliable indicator of a true intrauterine pregnancy. Hyperdecidual reaction pseudo-sacs often have irregular shapes and lack an echogenic rim that surrounds a true gestational sac. Careful examination for a trilaminar endometrium pattern is more suggestive of a non-pregnant state. The use of Doppler may also help as a pseudo-sac will not have enhanced vascularity around it.

In the index case, laparoscopic resection of the ectopic pregnancy was done due to the high risk of rupture. Dilatation and evacuation of the non-viable intrauterine pregnancy were performed at the same time. However, the histopathological report confirmed it as an ectopic pregnancy with an intrauterine hyperdecidual reaction.

## Conclusions

The diagnosis and management of heterotopic pregnancy, hyperdecidual reaction, and ectopic gestation represent a formidable challenge in obstetrics and gynecology. However, with a thorough understanding of the underlying pathophysiology, combined with a multidisciplinary approach to diagnosis and management, this diagnostic maze can be navigated with confidence. By prioritizing early recognition and timely intervention, we can mitigate the risks associated with these conditions and optimize outcomes for patients and their families. Radiological diagnosis plays an important role in the early gestational age. It is important to note that early diagnosis and intervention are critical in managing heterotopic pregnancies to reduce the risk of complications. Women who have undergone fertility treatments such as in vitro fertilization (IVF) are at a higher risk of experiencing heterotopic pregnancies, so close monitoring during the early stages of pregnancy is especially important in such cases.

## References

[1] Sivalingam VN, Duncan WC, Kirk E, Shephard LA, Horne AW (2011). Diagnosis and management of ectopic pregnancy. J Fam Plann Reprod Health Care.

[2] Talbot K, Simpson R, Price N, Jackson SR (2011). Heterotopic pregnancy. J Obstet Gynaecol.

[3] Oancea M, Ciortea R, Diculescu D (2020). Spontaneous heterotopic pregnancy with unaffected intrauterine pregnancy: systematic review of clinical outcomes. Medicina (Kaunas).

[4] Mj G, R R (2008). Heterotopic pregnancy in natural conception. J Hum Reprod Sci.

[5] Barrenetxea G, Barinaga-Rementeria L, Lopez de Larruzea A, Agirregoikoa JA, Mandiola M, Carbonero K (2007). Heterotopic pregnancy: two cases and a comparative review. Fertil Steril.

[6] Michał M, Marian M, Marek M, Ewa WO (2011). Heterotopic pregnancy in the absence of risk factors--diagnostics difficulties. Ginekol Pol.

[7] Mohapatra I, Samantray SR (2021). Scar ectopic pregnancy - an emerging challenge. Cureus.

[8] Lavanya R, Deepika K, Patil M (2009). Successful pregnancy following medical management of heterotopic pregnancy. J Hum Reprod Sci.

[9] Ramalho I, Ferreira I, Marques JP (2019). Live birth after treatment of a spontaneous ovarian heterotopic pregnancy: a case report. Case Rep Womens Health.

[10] Barnhart KT, Guo W, Cary MS (2016). Differences in serum human chorionic gonadotropin rise in early pregnancy by race and value at presentation. Obstet Gynecol.

[11] Fernandez H, Capmas P, Lucot JP, Resch B, Panel P, Bouyer J (2013). Fertility after ectopic pregnancy: the DEMETER randomized trial. Hum Reprod.

[12] Jan F, Naikoo GM, Rather MH, Sheikh TA, Rather YH (2010). Ruptured heterotopic pregnancy: a rare cause for hemoperitoneum; report of three cases from kashmir, India. Indian J Surg.

[13] Onoh RC, Ejikeme BN, Onwe AB, Asiegbu OU (2018). Ruptured ectopic in heterotopic pregnancy: management and spontaneous vertex delivery of a live baby at term. Niger J Clin Pract.

[14] Allison JL, Aubuchon M, Leasure JD, Schust DJ (2012). Hyperosmolar glucose injection for the treatment of heterotopic ovarian pregnancy. Obstet Gynecol.

[15] Goldstein JS, Ratts VS, Philpott T, Dahan MH (2006). Risk of surgery after use of potassium chloride for treatment of tubal heterotopic pregnancy. Obstet Gynecol.

[16] Martin JA, Hamilton BE, Osterman MJ (2012). Three decades of twin births in the United States, 1980-2009. NCHS Data Brief.

